# Twenty years of Colombian experience with enzymatic screening in patients with features of mucopolysaccharidosis

**DOI:** 10.1002/jmd2.12313

**Published:** 2022-07-28

**Authors:** Alfredo Uribe‐Ardila, Johana Ramirez‐Borda, Adis Ayala

**Affiliations:** ^1^ Faculty of Sciences, Biochemistry Research Center Universidad de Los Andes Bogota Colombia; ^2^ Faculty of Sciences and Education, Biochemistry and Molecular Biology Group Universidad Distrital Francisco José de Caldas Bogota Colombia

**Keywords:** arylsulfatase B, dried blood spots, enzymatic diagnosis, iduronate 2‐sulfatase, lysosomal disorders, mucopolysaccharidosis, *N*‐acetylglucosamine‐6‐sulfate sulfatase, α‐*N*‐acetylglucosaminidase, α‐l‐iduronidase, β‐galactosidase, β‐glucuronidase

## Abstract

Mucopolysaccharidoses (MPSs) are a group of genetic alterations whose effect is the progressive intralysosomal accumulation of glycosaminoglycans. Affected individuals are deficient in one or more lysosomal enzymes which, depending on the MPS, may cause coarse facial features, short stature, multiple skeletal dysplasia, joint stiffness, or developmental delay. Their diagnosis is mostly performed late or incorrectly, and it represents a challenge since it requires specialized tests only performed in major cities. This makes it difficult for patients to have access to physicians since their geographical location is distant and therefore, the use of samples collected in solid‐phase represents an advantage for the study of high‐risk populations. In addition, epidemiological information about rare diseases, especially in Latin America, is scarce or inconsistent. Our aim was to report the experience of 20 years of selective screening by assessing enzyme activity and reporting incidence values of MPS in Colombia. This study validated a group of fluorometric endpoint techniques in 8239 patients. The samples were dried blood spots (DBS) collected on filter paper and leukocyte extracts. Reference values in the Colombian population for α‐l‐iduronidase, iduronate 2‐sulfatase, α‐*N*‐acetylglucosaminidase, *N*‐acetylglucosamine‐6‐sulfate sulfatase, β‐galactosidase, arylsulfatase B, and β‐glucuronidase were established in leukocyte extracts, and patients reference ranges were updated in the case of DBS samples. Incidence values were calculated for each MPS and the distribution of cases across the country is also shown. This study offers very useful information for the health system, the scientific community, and it facilitates the diagnosis of these disorders. This is indispensable when seeking to develop new diagnostic or treatment approaches for patients.

## 1 INTRODUCTION

Mucopolysaccharidoses (MPSs) are a type of lysosomal storage disorder caused by the hereditary deficiency of lysosomal enzymes necessary in the degradation of glycosaminoglycans (GAGs).^1^ General symptoms of these disorders include dysostosis multiplex, coarse facial features, and cardiovascular and respiratory conditions.^2^ Body symptoms also include short stature, skeletal dysplasia, visceromegaly, and joint stiffness.^3^, ^4^, ^5^

The diagnosis of MPS is mostly performed late or incorrectly for prolonged periods^6^ and patients often must see many medical specialists before being correctly diagnosed.^7^ Schieppati et al.^8^ found that in 25% of cases, ~5–30 years elapse between the appearance of the first symptoms and the delivery of an accurate diagnosis. Furthermore, 40% of the patients reported being subjected to inappropriate medical treatment (33%) or even surgery (16%), events to which MPS patients and families have been exposed as well. Laboratory tests for diagnosis of MPS usually involve the use of liquid samples (whole blood or leukocyte extracts^9^); however, these specimens require strict conditions of conservation, packaging, and referral time.

It is known that the epidemiological information available on rare diseases is scarce or inconsistent among different sources.^8^ Some studies have proposed that rare diseases, especially in Latin America, may be under‐diagnosed and therefore the estimated frequencies may be erroneous. A study conducted in Colombia estimates underdiagnosis due to the lack of trained medical personnel, the variety of clinical manifestations, and the absence of a reference center for the diagnosis of these diseases.^10^

This study was aimed at establishing reference values in the Colombian population using the gold standard method in leukocytes for the following enzymes: α‐l‐iduronidase (IDUA) (EC3.2.1.76), iduronate 2‐sulfatase (IDS) (EC3.1.6.13), α‐*N*‐acetylglucosaminidase (NAGLU) (EC3.2.1.50), *N*‐acetylglucosamine‐6‐sulfate sulfatase (GALNS) (EC3.1.6.14), β‐galactosidase (GLB) (EC3.2.1.23), arylsulfatase B (ARSB) (EC3.1.6.1), and β‐glucuronidase (GUSB) (EC3.2.1.31). Also, to update reference ranges in the dried blood spot (DBS) technique since we studied a larger population than in previous studies.^18^ Regarding MPS incidence, to calculate the corresponding values, and illustrate their distribution across the Colombian territory. Our main goal was to increase the rate of accurate and early diagnosis, so the patients get into therapy as early as possible.

## 2 MATERIALS AND METHODS

### 2.1 Sampling and reagents

DBS impregnated on filter paper from 519 healthy individuals and 8239 patients with clinical suspicion of MPS were analyzed from 2000 to 2020. All individuals consented autonomously and signed an informed consent form. Procedures followed the Declaration of Helsinki of 1993 and were approved by the ethics committee of the Universidad de Los Andes. The filter paper used for whole‐blood collection was grade 903 provided by Schleicher and Schuell, Whatman®. The blood spots were dried at room temperature for 8–12 h and then were stored at 4°C in self‐sealing plastic bags. The maximum time elapsed between sample collection and the analytical process did not exceed 30 days.

The fluorogenic substrates used for the filter paper and leukocyte assays are shown in Tables S1 and s2. The assays were performed in 96‐well black polypropylene microplates, using aluminum foil heat‐sealing foils (Corning‐Lowell, USA). The elution process required a Titramax1000 vibrator and plate shaker. Incubation with orbital shaking was performed in the Unimax1010 incubator/stirrer, both provided by the Heidolph Group. A SpectramaxM2 from Molecular Devices Corp. was used as a fluorescence reader.

### 2.2 Enzyme activity assays in DBS and leukocytes

The assays in DBS were adapted from Chamoles et al.,^11^ Civallero et al.,^12^ Voznyi et al.,^13^ and Ceci et al.,^14^ and these methodologies were modified to implement a 1.2 mm diameter punch (~0.52 μl of blood). The leukocyte analyses were performed using as a reference the methods described in Shapira et al.^15^ and Diggelen et al.^16^ Adjustments were also applied, except for the method described in Voznyi et al.^13^ (Table S2). The protein concentration was assessed according to Lowry et al.^17^ and BCA assay (Thermo Fisher). Evaluation of all samples was performed in triplicate and, GLB activity was quantified to assess their quality. When a patient was found to have deficient enzymatic activity in DBS, both blood and urine samples were required to confirm this diagnosis through the electrophoretic analysis of GAGs and the gold standard technique.

### 2.3 Statistical analysis

Descriptive statistics and graphical representation of the data were performed using the IBM SPSS 19 Statistical Package, Microsoft Excel software (Microsoft Corporation), and Paintmaps.com (ColorfulMaker.com). The incidence calculation was performed based on statistics from DANE (National Administrative Department of Statistics) Colombia.

## 3 RESULTS AND DISCUSSION

Out of the 8239 samples submitted to our laboratory over 20 years, we found 370 patients to be affected and their corresponding enzyme activities gave rise to the reference ranges shown in Tables 1 and 2. All patients showed control enzyme activities comparable to those reported for healthy individuals in the Colombian population (data not shown).

**TABLE 1:** Results of enzyme activity in DBS from controls and patients

Deficient enzyme	*n* affected/total control	Activity (nmol/ml/h) range—average, SD
IDUA (age range: 0.1–27.8 years)	37/8239 studied	0.00–0.8 A: 0.2, SD: 0.2
Controls (3 months–59 years)^a^	1585^a^	1.5–20.1 A: 9.5, SD: 3.8^a^
IDS (age range: 0.7–32.2 years)	40/600 studied	0.00–2.1 A 1.1, SD: 0.4
Controls (3 months–59 years)^a^	210^a^	10.7–45.2 A: 24.8, SD: 7.3^a^
NAGLU (age range: 0.8–16.7 years)	14/409 studied	0.10–0.9 A: 0.5, SD: 0.3
Controls (1 month–58 years)	263	2.8–9.4 A: 0.5, SD: 0.3
β‐Galactosidase (age range: 0.5–51.4 years)	9/8239 studied	0.00–5.2 A: 2.4, SD: 1.4
Controls (3 months–88 years)^a^	2354^a^	19–99 A: 47, SD: 26^a^
ARSB (age range: 0.3–24.3 years)	53/8239 studied	0.00–2.3 A 0.8, SD: 0.7
Controls (3 months–59 years)^a^	625^a^	2.9–43.2 M: 9.2, SD: 5.6^a^
GUSB (age: 2 months)	1/2813 studied	0.18
Controls (4 months–76 years)^b^	971^b^	31.2–242.6 M: 112.8, SD: 43^b^

^a^ Adapted from Uribe et al.^18^

^b^ Adapted from Uribe et al.^20^ (these values are shown only to compare the affected and control population).

**TABLE 2:** Reference values and cutoff points obtained in the enzyme studies in leukocytes

Deficient enzyme^a^	*n* affected/total control	Activity (nmol/ml/h) range—average, SD	Residual activity %
IDUA (age range: 0.1–27.8 years)	37/8239 studied	0.0–1.3 A: 0.3, SD: 0.3	11.8
Controls (30 days–75 years)	519	3.9–55.0 A: 13.2, SD: 7.9	
IDS (age range: 0.7–32.2 years)	40/600 studied	0.00–4.9 A: 0.7, SD: 0.8	21.7
Controls (4 months–75 years)	94	7.5–55.1 A: 25.1, SD: 11.3	
NAGLU (age range: 0.8–16.7 years) Controls (6 months–70 years)^b^	14/409 studied	0.01–0.3 A: 0.2, SD: 0.1	18.5
	463^a^	0.6–4.0 A: 1.7, SD: 0.7^a^	
GALNS (age range: 2 days to 57.3 years) controls (30 days–82 years)	216/2500 studied	0.00–0.5 A: 0.06, SD: 0.09	7.2
	302	2.5–17.9 A: 7.0, SD: 2.4	
GLB (age range: 0.5–51.4 years)	9/8239 studied	1.4–6.0 A: 4.7, SD: 1.5	2.9
Controls (2 months–75 years)^c^	1492^b^	80.1–557 A: 222.4, SD: 99.4^b^	
ARSB (age range: 0.3–24.3 years)	53/8239 studied	0.0–68.7 A: 18.1, SD: 15.3	34.5
Controls (30 days–75 years)	250	110.2–425.6 A: 216.8, SD: 80.7	
GUSB (age: 2 months)	1/2813 studied	0.35	0.1
Controls (15 days–65 years)	300	100.1–578.7 A: 273.8, SD: 98.5	

^a^ Mann–Whitney test between controls and affected patients (*p* < 0.0001).

^b^ Adapted from Ramirez Borda and Uribe.^24^

^c^ Adapted from Uribe et al.^25^ (these values are shown only to compare the affected and control population).

### 3.1 Enzymatic studies in DBS

One hundred percent of the enzyme activities of the affected patients fall between the previously reported ranges and the cutoff point reported by Uribe et al.^18^ and Bender et al.^19^ This fact allowed us to corroborate their diagnosis as affected and to propose the standardized protocols for DBS as a screening tool.

It should be noted that DBS results depend on the impregnation and drying of the filter paper. We recommend quantifying the activity of one or two control enzymes to guarantee the integrity of the sample and to re‐evaluate in a new sample when inconclusive or positive cases are found. Several studies recommend performing a second confirmatory test either by GAG quantification or enzymatic tests on DBS and leukocytes to ensure an accurate diagnosis and avoid false positives.^21^, ^22^

Another important aspect is the fact that a low leukocyte count present in these individuals can notably affect the results of the enzymatic test. Such phenomenon has been previously studied by Chamoles et al.,^11^ who found a positive correlation between the level of enzyme activity on DBS and the total leukocyte count in blood. Sözmen et al.^23^ found that, when recalculating the glucocerebrosidase activity before and after taking into account the leukocyte count, the false positives in the DBS test decreased from 53 to 12.

### 3.2 Enzymatic studies in leukocytes

Although the DBS methodology usually correctly discriminates healthy individuals from affected individuals, it is intended for screening purposes only and is therefore not a definitive diagnostic test. On the contrary, it requires positive cases to be corroborated with the gold standard, which consists of enzymatic quantification in cell extracts. In this study, the following enzymes activity reference range in leukocytes was established: IDUA, IDS, NAGLU, GALNS, GLB, ARSB, and GUSB (Table 2). Also, a total of 519 controls with an age range of 30 days to 82 years were analyzed.

The percentage of residual activity of affected patients ranges between 0% and 34%, compared with the median of the control individuals (Table 2). There is a greater discriminatory power in the GLB assay since the maximum residual activity in affected individuals was only 2.97%. This value is in agreement with that previously found by Yuskiv et al.^26^ who reported a range of 2%–11.5% for patients with skeletal manifestations only, and a range of 4.6%–14.1% for patients with severe clinical features. Uribe et al.^20^ reported a maximum residual activity value of 12.41%.

On the other hand, ARSB showed the highest residual activity (34.4%). This is not the first time that a value of this magnitude is reported for this enzyme since Uttarilli et al.^27^ demonstrated that cells transfected with clones having the p.His393Arg and p.Trp450Leu had residual activities of 38% and 41%, respectively. In fact, this same study found a genotype–phenotype correlation for these two variants since the intermediate clinical presentation was associated with approximately half of the residual activity compared with other mutations. However, it should be clarified that this correlation has not been found for all mutations and that in most cases enzyme activity is not clearly related to the phenotype of patients with MPS VI.^28^, ^29^

### 3.3 Distribution and incidence of MPS in Colombia

**FIGURE 1:**
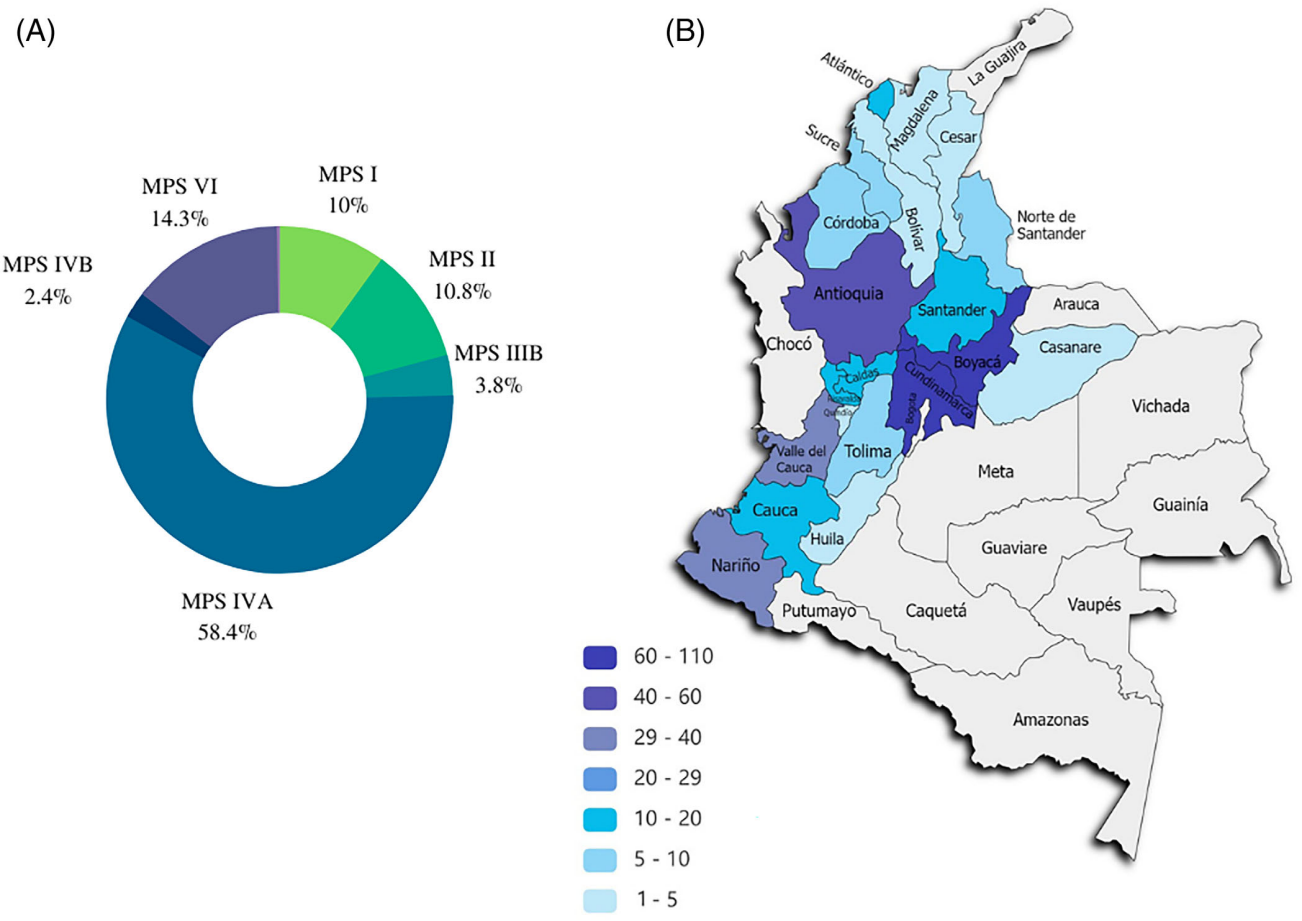
(A) Numerical proportion of MPS cases in relation to the 370 positive patients found in this study. (B) Region of origin of the positive patients found in this study

About 4.5% of patients who were referred to our laboratory over 20 years suffer from MPS. The analysis of these patients yielded the following distribution of MPS cases: 37 resulted deficient for IDUA, 40 for IDS, 14 for NAGLU, 216 for GALNS, 9 for GLB, 53 for ARSB, and 1 for GUSB (Figure 1A). Assuming this percentage as the total of affected patients, the frequency of occurrence in ascending order is as follows: MPS VII, MPS IVB, MPS IIIB, MPS I, MPS II, MPS VI, and MPS IVA, where the last is the most frequent among the Colombian population at 58.4%.

When looking for previously reported information, there is a high heterogeneity regarding which is the most frequent MPS in each country. For example, MPS II was the most frequent in Asia according to Lin et al.,^30^ Chen et al.,^31^ Cho et al.,^32^ and Khan et al.,^33^ who reported values between 47.4% and 58.1% in the countries of Taiwan, China, South Korea, and Japan, respectively. A study conducted in the United States^34^ reported that MPS III was the most frequent at 26.6%, followed by MPS I and II at 26.23%.

As shown in Figure S1, the number of analyzed samples for each of the studied enzymes was not always the same. Although this study lasted a total of 20 years, not all the techniques mentioned here were standardized at the same time. The first enzymatic techniques correspond to MPS I, IVB, and VI, which were implemented since 2000. Second, the techniques for MPS II and IVA were established in the year 2010, and the techniques for MPS IIIB and VII in the years 2015 and 2017, respectively. This graph shows the percentage of affected individuals for a given MPS, only regarding the number of samples with which it was analyzed, and not the total number of affected individuals. When analyzing this graph, we observe that MPS IVA continues to have the highest proportion of positive cases, which is consistent with the findings of a previous study conducted by Gómez et al.^10^ in two departments of Colombia (12 cases out of 35).

Figure 1B shows the distribution of the MPS positive cases found in this work, most of them belong to the Cundinamarca‐Boyacá highlands with 114 cases, followed by Antioquia with 56 cases. The high percentages of patients found in these departments (31% and 15%, respectively) are consistent with the expected since they come from the closest regions to the capital of the country. Therefore, patients have more possibilities of being transferred to a medical center or sending their samples to a nearby laboratory under optimal conservation and time conditions. Gómez et al.^10^ suggested consanguinity as a cause for the high presence of MPS in Boyacá. Pacheco‐Orozco et al.^35^ analyzed the high incidence of rare diseases in this department, finding that there is indeed an increased inbreeding rate and therefore this population may be genetically isolated.

The Southern region of the Colombian Pacific, composed of Nariño, Cauca, and Valle del Cauca, together account for 83 cases (22%). It is noteworthy that even though these regions are located at the very border of the country, they still present such a high rate of referral and positive cases. To date, no MPS studies have been found in this region or in any other region different from the Cundiboyacense highlands in Colombia. It would be worthwhile to conduct more enzymatic, epidemiological, and genetic studies in this region since the presence of consanguinity should not be ruled out as a risk factor. This phenomenon has been previously reported in countries such as Egypt,^36^ Tunisia,^37^, ^38^ and Brazil.^39^

Table 3 shows that MPS VII had the lowest incidence values in all cases. These observations agree with what is found in the literature, since it is considered an ultra‐rare disease, with a prevalence of <1:1 000 000.^41^ The incidence value found in our study is higher than that reported in the United States and Taiwan but is similar to that previously reported by Zielonka et al.^42^ of 0.01.

**TABLE 3:** Incidence of MPS according to the number of live births in the study period in Colombia

	Incidence (affected/100 000 live births)					
	Colombia	The United States^a^	Sweden^b^	Norway^b^	Denmark^b^	Taiwan^c^
MPS I	0.26	0.26	0.67	1.85	0.54	0.11
MPS II	0.56	0.26	0.27	0.13	0.27	1.07
MPS IIIB	0.36	0.05	0.67 (III)	0.27 (III)	0.43 (III)	0.28
MPS IVA	3.00	0.11	0.07 (A and B)	0.76 (A and B)	0.48 (A and B)	0.33
MPS IVB	0.06	0.004				0
MPS VI	0.37	0.04	0.07	0.07	0.05	0.14
MPS VII	0.04	0.0027	NA	NA	NA	0

Abbreviation: NA, no information was reported in the corresponding studies.

^a^ Adapted from Puckett et al.^34^

^b^ Adapted from Malm et al.^40^

^c^ Adapted from Lin et al.^30^

The incidence of MPS IVA was higher than that of MPS IVB in all cases. This is consistent with the reported information by the NORD (National Organization for Rare Disorders), who estimates that 95% of the cases of Morquio disease belong to type A. In Colombia, 216 cases were detected throughout 10 years of study, when ~7 204 000 live births were reported. These data yielded an incidence of three affected persons per 100 000 live births. This value is considerably higher than those previously reported for MPS IVA and the closest one was found in Northern Ireland with a value of 1.32.^43^ Other high incidence values for MPS have been reported for MPS I and III in Northern Ireland and Australia (3.8^43^ and 1.72,^44^ respectively).

The high incidence of MPS IVA in our country could be explained by the fact that the phenotype of most patients is usually severe or at least easily recognizable and it is the most common form.^45^ This may cause physicians to recognize it easily and therefore refer patients for biochemical tests more efficiently. Puckett et al.^34^ proposed that countries with populations that tend to be encapsulated and without a high rate of migration and genetic exchange could concentrate more mutations that give rise to this group of diseases. This phenomenon has influenced the MPS incidence in countries such as Northern Ireland where a high proportion has been seen in the Irish Traveler population who tend to intermarry, and in Saudi Arabia where this situation has caused a high incidence of 16.9.^34^

It is worth noting that the population that reached the final confirmation process in the present investigation ranged from 8 months to 35 years, and only 9% were younger than 2 years. This situation suggests a late diagnosis, often resulting in irreversible implications that usually cannot be ameliorated. Finally, we recommend studying a sample of carriers in the future to accurately determine the behavior of the enzyme in this population and to develop effective enzyme assays that can discriminate carriers from noncarriers.

## 4 CONCLUSIONS

The phenotypic variety of the MPS together with the epigenetics of each individual and the difficulty in establishing an accurate diagnosis make biochemical assessment a useful tool in the screening of populations with these metabolic disorders. Our study focused on optimizing a set of methodologies for enzymatic analysis in DBS that reduce the volume of reagents and sample used and the processing time compared with other methods. This is not only to maximize the use of the specimens submitted but also to reduce processing costs and to provide a rapid emission of results. This study provides the validation of 14 micro techniques observed in Tables S1 and S2 with their respective reference ranges of activity for seven enzymes in a Colombian population sample.

The findings of 20 years of work constitute a record that has no precedent in the existing documentation in Colombia on rare disorders. We aimed to raise awareness, increase early detection, and to provide a correct orientation to affected families. Under the implementation of these methodologies and the reporting of incidence values, our hope is to alert the health system and to expand the research capacity around these diseases. Studies like this are indispensable to carry out neonatal screening research and to perform cost‐benefit calculations when new diagnostic or treatment methods are to be developed.

## CONFLICT OF INTEREST

The authors declare that there is no conflict of interest that could be perceived as prejudicing the impartiality of the research reported.

## ETHICS STATEMENT

All procedures followed were in accordance with the ethical standards of the responsible committee on human experimentation (Universidad de Los Andes) and with the Helsinki Declaration of 1993. Informed consent was obtained from all patients being included in the study. In addition, no personal information about any patient was disclosed (proof that informed consent was obtained is available upon request).

## ANIMAL RIGHTS

This article does not contain any studies with animal subjects performed by any of the authors.

## Supporting information


**Appendix S1.** Supporting Information.

## Data Availability

All original data supporting the reported results can be obtained by contacting the corresponding author.
