# Supplementary material for: Twenty years of Colombian experience with enzymatic screening in patients with features of mucopolysaccharidosis
Source: JIMD Rep. 2022 Jul 28;63(5):475–83. doi: 10.1002/jmd2.12313 (PMC9458599; doi:10.1002/jmd2.12313)
Supplement: Supplementary file 1 — Appendix S1. Supporting Information. [file JMD2-63-475-s001.docx]

**Supplementary Material**

**Table S1.** Conditions of the enzymatic assays evaluated on filter paper.

| **Enzyme** | **Reaction buffer** | **Substrate * (Concentration in the reaction buffer)** | **Incubation time/°C** | **Stop buffer** |
| --- | --- | --- | --- | --- |
| **α – L -**  **Iduronidase** | 20 µL of 0.2 M sodium bromate buffer, pH 2.8 with 10 µL of 3.1 mM D-saccharolactone | 4-Methylumbelliferyl α -L- iduronide 2 mM | 20 h/  37°C | 200 µL Glycine carbonate |
| **Iduronate 2-sulfatase** | ***Pre-incubation:*** place each 1.2 mm punch in 25 µL of 0.2% BSA ***Incubation 1:*** add 16 µL of 0.1 M acetate buffer, pH 5.0 with 10 mM lead acetate and substrate at 1.25 mM  ***Incubation 2:*** Add 25 µL of 0.2 M citrate-phosphate buffer, pH 4.5 with 0.02% sodium azide and auxiliary enzyme | ***Incubation 1***: 4-Methylumbelliferyl α -iduronate-2-sulfate 1.25 mM  ***Incubation 2***: enzyme α -L-iduronidase 6.4 mg/mL in distilled water | ***Incubation 1*:**  24 h/37°C  ***Incubation 2*:**  24 h/37°C | 200 µL Glycine carbonate  (Add at the end of the second incubation) |
| **α -N -acetylglucosaminidase** | 30 µL citrate-phosphate buffer 0.2 M, pH 4.3 | 4-metilumbeliferil-2-acetamido-2-deoxy-α-D-glucopyranoside in distilled water 2.5 mmol/L | 20 h/  37°C | 150 µL Glycine carbonate |
| **β -Galactosidase** | 40 µL de citrate-phosphate buffer 0.1 M, pH 4.4 with 20 µL NaCl 0.15 M | 4-Methylumbelliferyl -D-Galactoside 0.8 mM | 3 h/  37°C | 200 µL Glycine carbonate |
| **Arylsulfatase B** | 40 µL acetate buffer 0.5 M, pH 6.0 with 10 mM barium acetate | 4-Methylumbelliferyl Sulfate 5.5 mM | 24 h/37°C | 150 µL Glycine carbonate |
| **β -Glucuronidase** | 30 µL acetate buffer 0.1 M, pH 4.8 | 4-Methylumbelliferyl β-D-glucuronide dihydrate 10 mM | 2 h/37°C | 150 µL Glycine carbonate |

*All substrates were supplied by Sigma Co. (St. Louis, MO, USA) except for the iduronate 2-sulfatase assay auxiliary enzyme substrate (Moscerdam Substrates. Rotterdam, the Netherlands) and the α-iduronate enzyme substrate (Toronto Research Chemicals. Toronto, Canada).

**Table S2.** Conditions of the enzymatic assays in leukocytes.

| **Enzyme** | **Reaction buffer** | **Substrate (Concentration in the Reaction buffer)** | **Incubation time/°C** | **Stop buffer** |
| --- | --- | --- | --- | --- |
| **α – L Iduronidase** | 50 µL citrate-phosphate buffer 0.5 M, pH:4.3 with D-saccharolactone 3 mM and 50 µL leukocyte lysate* | 4-Methylumbelliferyl α -L-iduronide 5 mM | 1 h/ 37°C | 650 µL Glycine carbonate |
| **Iduronate 2-sulfatase** | 5 µL leukocyte lysate** and 5 µL of BSA 0.2%  ***Incubation 1:*** add 16 µL of 0.1 M acetate buffer, pH 5.0 with 10 mM lead acetate.  ***Incubation 2:*** add 16 µL of 0.2 M citrate-phosphate buffer, pH 4.5 with 0.02% sodium azide and auxiliary enzyme | ***Incubation 1:*** 4-Methylumbelliferyl α -iduronate-2-sulfate 1.25 mM  ***Incubation 2:*** α – L- iduronidase 6.4 mg/mL in 8 ul distilled water | ***Incubation 1:*** 4 h/37°C  ***Incubation 2:*** 24 h/37°C | 200 µL Glycine carbonate  (Add at the end of Incubation 2) |
| **α-N -Acetylglucosaminidase** | 50 µL Citrate phosphate 0.2 M, pH 4.3 con 50 µL de leukocyte lysate* | 4-Methylumbelliferyl-2-acetamido-2-deoxy-α -glucopyranoside 2.5mM | 2 h/  37°C | 650 µL de Glycine carbonate |
| **N-Acetyl- galactosamina-6-sulfato sulfatase** | 10 µL leukocyte lysate** and 10 µL of BSA 0.2%  ***Incubation 1:*** add 20 µL of acetate buffer 0.1 M, pH 4.3, NaCl 0.1M.  ***Incubation 2***: add 8 µL of 0.9 M sodium phosphate buffer, pH 4.3 with 0.02% sodium azide and 10 µL of auxiliary enzyme β-Galactosidase. | ***Incubation 1:*** 4-Methylumbelliferyl B-D-galactopyranoside-6-sulphate sodium salt 10mM ^a^  ***Incubation 2:*** β -Galactosidase 1 mg/mL in distilled water | ***Incubation 1:*** 17 h/37°C  ***Incubation 2:*** 2h/37°C | 200 µL Glycine carbonate  (Add at the end of incubation 2) |
| **β -Galactosidase** | 50 µL citrate-phosphate buffer 0.1 M, pH 4.4 with 5.84 mg/mL NaCl and 50 µL leukocyte lysate* | 4-Methylumbelliferyl -D-Galactoside 0.8 mM | 20 min/ 37°C | 650 µL Glycine carbonate |
| **Arylsulfatase B** | 100 µL de acetate buffer 0.5 M, pH 6.0 con Acetato de Bario 10 mM y 100 µL leukocyte lysate * | 4-Nitrocatechol sulfate 50 mM | ***Incubation 1:*** 30 min /37°C ***Incubation 2:*** 1.5 h /37°C | 300 µL NaOH 0.1 M |
| **β -Glucuronidase** | 50 µL of 0.1 M acetate buffer, pH 4.8 and 50 µL of leukocyte lysate* | 4-Methylumbelliferyl β-D-glucuronide dihydrate 10 mM | 20 min/ 37°C | 650 µL Glycine carbonate |

Average protein value in mg in the indicated sample volume: * 0.05 mg / ** 0.01 mg.


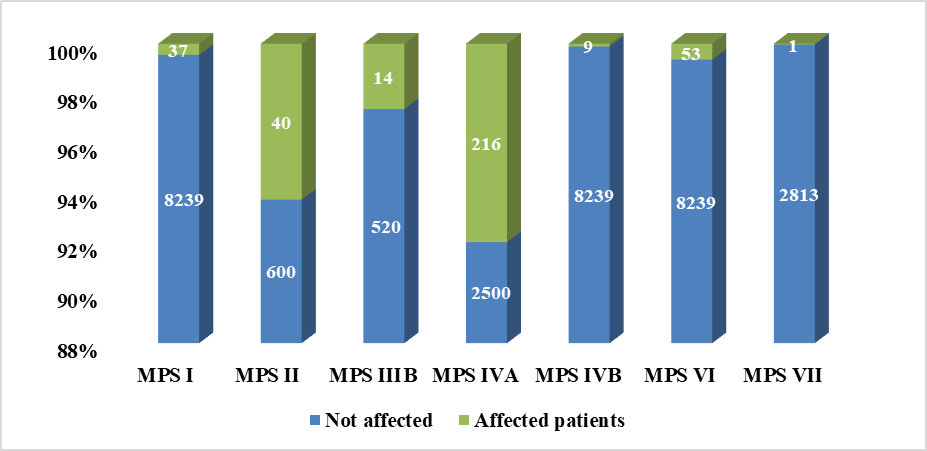


**Figure S1**. Proportion of affected individuals according to the number of samples analyzed for each MPS.
